# The Efficacy of Triptolide in Preventing Diabetic Kidney Diseases: A Systematic Review and Meta-Analysis

**DOI:** 10.3389/fphar.2021.728758

**Published:** 2021-10-01

**Authors:** Dongning Liang, Hanwen Mai, Fangyi Ruan, Haiyan Fu

**Affiliations:** ^1^ State Key Laboratory of Organ Failure Research, National Clinical Research Center of Kidney Disease, Guangdong Provincial Key Laboratory of Renal Failure Research, Division of Nephrology, Nanfang Hospital, Southern Medical University, Guangzhou, China; ^2^ The First Medical College, Southern Medical University, Guangzhou, China

**Keywords:** triptolide, renin-angiotensin system inhibitor, diabetic kidney diseases, albuminuria, kidney function

## Abstract

**Ethnopharmacological Relevance:** Triptolide (TP), the primary biologically active ingredient of *Tripterygium wilfordii Hook F* (TWHF), possesses the potential to solve the shortcomings of TWHF in treating diabetic kidney disease (DKD) in the clinic.

**Aim of the Study:** We conducted a meta-analysis to evaluate the efficacy of TP in treating DKD and offer solid evidence for further clinical applications of TP.

**Materials and Methods:** Eight databases (CNKI, VIP, CBM, WanFang, PubMed, Web of Science, EMBASE, and Cochrane library) were electronically searched for eligible studies until October 17, 2020. We selected animal experimental studies using TP *versus* renin–angiotensin system inhibitors or nonfunctional liquids to treat DKD by following the inclusion and exclusion criteria. Two researchers independently extracted data from the included studies and assessed the risk of bias with the Systematic Review Centre for Laboratory Animal Experimentation Risk of Bias tool. Fixed-effects meta-analyses, subgroup analyses, and meta-regression were conducted using RevMan 5.3 software. Inplasy registration number: INPLASY2020100042.

**Results:** Twenty-six studies were included. Meta-analysis showed that TP significantly reduced albuminuria (14 studies; standardized mean difference SMD: −1.44 [−1.65, −1.23], I^2^ = 87%), urine albumin/urine creatinine ratio (UACR) (8 studies; SMD: –5.03 [–5.74, −4.33], I^2^ = 84%), total proteinuria (4 studies; SMD: –3.12 [–3.75, −2.49], I^2^ = 0%), serum creatinine (18 studies; SMD: –0.30 [–0.49, −0.12], I^2^ = 76%), and blood urea nitrogen (12 studies; SMD: –0.40 [–0.60, −0.20], I^2^ value = 55%) in DKD animals, compared to the vehicle control. However, on comparing TP to the renin–angiotensin system (RAS) inhibitors in DKD treatment, there was no marked difference in ameliorating albuminuria (3 studies; SMD: –0.35 [–0.72, 0.02], I^2^ = 41%), serum creatinine (3 studies; SMD: –0.07 [–0.62, 0.48], I^2^ = 10%), and blood urea nitrogen (2 studies; SMD: –0.35 [–0.97, 0.28], I^2^ = 0%). Of note, TP exhibited higher capacities in reducing UACR (2 studies; SMD: –0.66 [–1.31, −0.01], I^2^ = 0%) and total proteinuria (2 studies; SMD: –1.18 [–1.86, −2049], I^2^ = 0%). Meta-regression implicated that the efficacy of TP in reducing DKD albuminuria was associated with applied dosages. In addition, publication bias has not been detected on attenuating albuminuria between TP and RAS inhibitors after the diagnosis of DKD.

**Systematic Review Registration:** https://clinicaltrials.gov/, identifier INPLASY2020100042

## Introduction

Diabetic kidney disease (DKD) is a chronic clinical condition characterized by micro- or macro-albuminuria followed by a progressive decline in kidney functions (Fu et al., 2019; Kopel et al., 2019). Over the past years, the incidence of DKD and its mortality has been largely underestimated (Rao et al., 2012). As a major driver of excess mortality in diabetes (Koye et al., 2018), DKD places growing financial burdens on diabetes management on a global scale (Slabaugh et al., 2015), especially in emerging and developing economies (Thomas et al., 2016). At present, antidiabetic medications and renin–angiotensin system (RAS) inhibitors are routinely used to prevent DKD from entering end-stage renal disease (ESRD) (Hostetter, 2001). However, this first-line therapy for DKD has been considered unsatisfying because of its potential side effects, such as diabetic ketoacidosis (Fadini et al., 2017) and reversible AKI (Onuigbo, 2011). Therefore, a systemic evaluation of the efficacy and safety of the current therapeutic strategies for DKD is urgently needed.

*Tripterygium wilfordii Hook F* (TWHF), a well-known Chinese herb, has been intensely developed and widely applied in treating nephritis or DKD in the clinic (Li et al., 2014; Huang et al., 2020; Xu et al., 2020; Guo Y et al., 2021). However, restricted by its adverse reactions and complex pharmacology (Hong et al., 2016; Ren et al., 2019; Huang et al., 2020), the extracts from TWHF have become a new focus in the field. The primary biologically active ingredient of TWHF, triptolide (TP), was discovered in 1972 (Kupchan et al., 1972). It is a striking target for total synthesis because of its intriguing structural features and promising biological activities (Zhang et al., 2019). TP can suppress inflammation and enhance cytoprotection by inhibiting the secretion of proinflammatory cytochemokines (Zhao et al., 2000; Zhou et al., 2003; Krakauer et al., 2005; Lu et al., 2005; Liu et al., 2006; Hoyle et al., 2010; Hou et al., 2019). Similar to TWHF, TP also induces organ or tissue damages (Xi et al., 2017), including hepatotoxicity (Li et al., 2014), nephrotoxicity (Yang et al., 2011), and reproductive toxicity (Ni et al., 2008). Along with advances in technology, TP exhibits great capacities in enhancing its efficacy, reducing side effects, and improving bioavailability through the nanostructured TP delivery system (Ren et al., 2021). Furthermore, the newly designed and synthesized water-soluble TP derivatives also demonstrated their safety and efficacy (Liu, 2011). These innovations significantly increased the application of TP in treating DKD. In this study, in contrast to RAS inhibitors and nonfunctional liquids, the primary objective is to systemically evaluate the efficacy of TP in animal models with DKD. Our analyses provide confidence for clinicians to design personalized therapeutic strategies for DKD under the current precision medicine model.

## Methods

This systematic review adhered to the preferred reporting items for systematic reviews and meta-analysis guidelines (Moher et al., 2009). The review protocol was registered in INPLASY before the beginning of the experiment (registration number: INPLASY2020100042).

### Publication Searching

We followed the Systematic Review Center for Laboratory Animal Experimentation (SYRCLE) step-by-step guide (Leenaars et al., 2012) to draw up the search strategy. Animal experimental studies of “TP treats DKD” were electronically searched in China National Knowledge Infrastructure (CNKI), Chinese Science and Technology Journal Database (VIP), Chinese Biomedical Database (CBM), WanFang, PubMed, Web of Science, EMBASE, and Cochrane library published from database inception to October 17, 2020. The search strategy consists of following three search components: triptolide AND DKD/diabetic nephropathy AND animals, using the Medical Subject Heading (MeSH) terms and keywords to perform searching. A pre-published animal filter (Hooijmans et al., 2010) was applied to limit the range for animal studies. All references of eligible articles were screened carefully for additional analyses.

### Inclusion and Exclusion Criteria

*Included criteria*: 1. Population: All animal models with DKD, regardless of species, age, or sex. 2. Intervention: The experimental groups used TP as monotherapy in any dosage. Comparison: The control groups received equal volumes of a nonfunctional liquid (normal saline) or did not receive treatment or recommended therapies according to clinical practice guidelines, including RAS inhibitors (angiotensin-converting enzyme inhibitors, ACEIs, or angiotensin AT (1)-receptor blockers, ARBs). 3. Study design: TP prepared in various forms, including extracts, granules, and injections, was eligible. 4. Outcome: The outcomes were changed in albuminuria, proteinuria excretion, and kidney function (serum creatinine, SCr, and blood urea nitrogen, BUN). 5. The literature is published either in Chinese or English.

*Excluded criteria*: 1. No *in vivo* studies (*in vitro* studies, clinical trials, review articles, case reports, comments, editorials, and abstracts). 2. Additional hypoglycemics were used during treatment. 3. Assessing TP combined with other herbal ingredients or complementary therapies. 4. Repeated literature. 5. Full text was not found.

### Data Extraction and Quality Assessment

Studies according to the inclusion criteria were considered eligible for the analyses. Data of each included study were extracted by two authors independently and cross-checked in terms of the author, publication year, animal models (species, sex, weight, method of modeling, and the criteria for successful modeling), TP intervention (the type of intervention, dosage, and the duration of intervention), and outcomes. When results were only reported graphically, the graph data were measured using GetData, software downloaded on the website (http://getdata.com.ru/). The total quality assessment of each study was evaluated based on the Systematic Review Centre for Laboratory Animal Experimentation Risk of Bias tool (Hooijmans et al., 2014). According to its guidance, each domain of individual studies was graded as low, unclear, or high risk of bias.

### Statistical Analysis

RevMan 5.3 software and Stata 15.1 software were used for data analysis. Studies were divided into two compilations to assess the differences between the TP and control groups and the differences between the TP and the RAS inhibitor groups. Continuous variables were expressed as standardized mean differences (SMDs) with a 95% confidence interval. A fixed effect was used in meta-analysis. Heterogeneity among the included studies was checked by using the chi-square test and I^2^ test. Substantial heterogeneity was considered to exist when I^2^ > 50%. Subgroup analyses and meta-regression were carried out to explore the possible sources of heterogeneity. High-risk studies were removed one by one from the group of the synthesized studies, and the remaining studies were re-analyzed to estimate the robustness of the results. Publication bias was evaluated by Begg’s test, Egger’s test, and the visual inspection of funnel plots for asymmetry.

## Results

### Search Results

**FIGURE 1:**
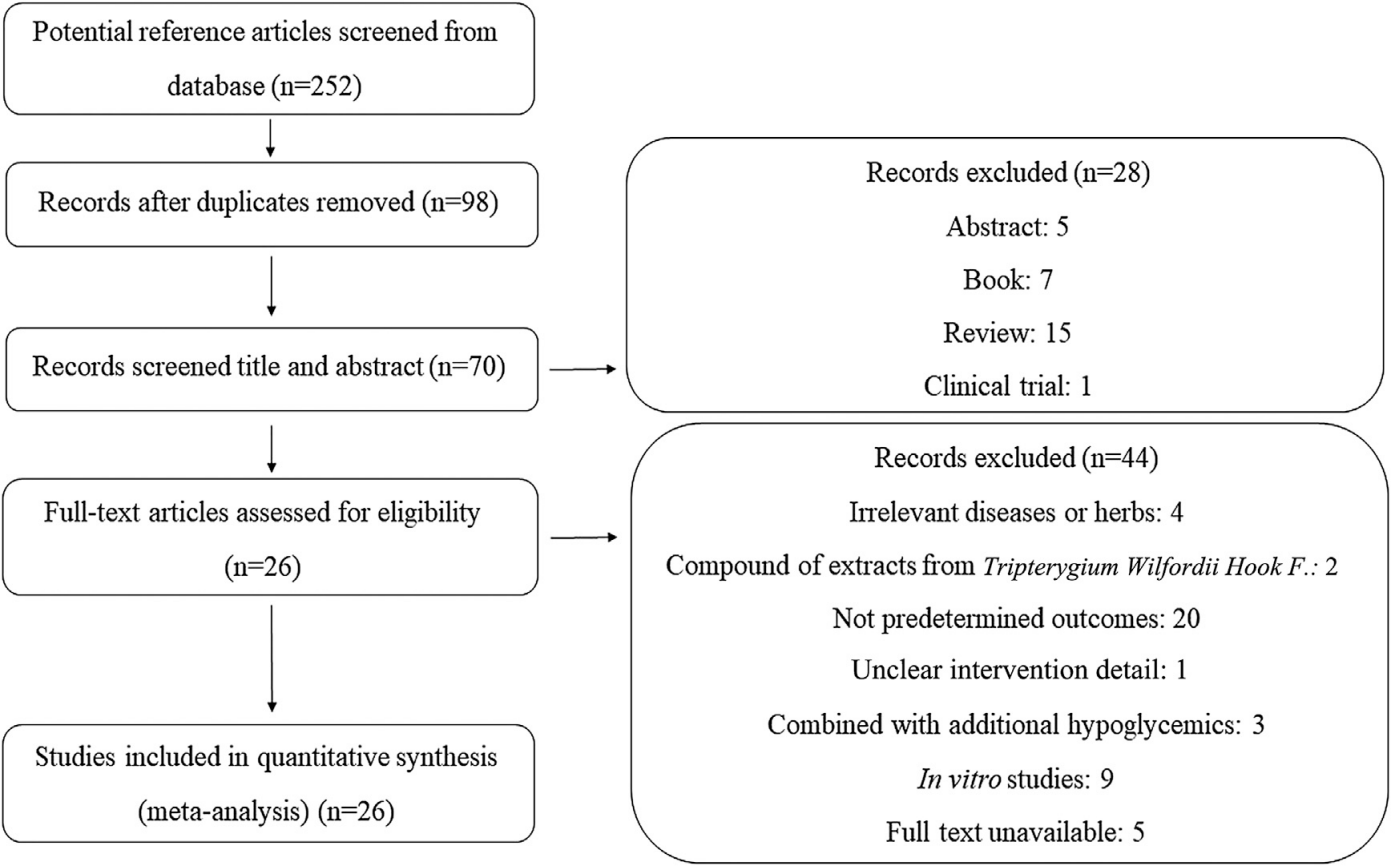
Flow chart of the screening process.

As illustrated in Figure 1, we identified 252 articles throughout the database. After removing duplications and screening the articles based on the titles and abstracts, the full texts of 70 studies were assessed for eligibility. Forty-four additional studies were excluded with the reasons being unclear intervention details and no predetermined outcomes, combining other extracts from TWHF or additional hypoglycemics for treatment, unavailable full texts, presenting *in vitro* experiments, and no relevance to diseases or herbs. Therefore, 26 studies were ultimately included in the analyses.

### Characteristics of the Included Studies

The details of the study characteristics are presented in Table 1. Twenty-six studies were published between 2008 and 2020. Five articles (Ma et al., 2013; Guo et al., 2016; Dong et al., 2017; Han et al., 2017; Li et al., 2017) were published in English, and the remaining 21 articles were published in Chinese. Four articles (Ma, 2009; Zhu, 2013; Han, 2018; Xue, 2018) were exhibited as dissertations, and the others were published in peer-reviewed journals. All research works tested the effects of TP on DKD, reporting at least one clinical parameter, including albuminuria, proteinuria, UACR, or kidney function. Among them, seven studies (Ma, 2009; Ma et al., 2009; Qao et al., 2009; Li et al., 2013; An et al., 2017; Dong et al., 2017; Ren et al., 2020) performed comparisons between TP and ACEI or ARB in treating DKD. Twenty-three studies used male Sprague–Dawley (SD) and Wistar rats, and the remaining three studies (Qao et al., 2009; Fan et al., 2018; Ren et al., 2020) used mutant or transgenic mice. The methods of modeling and the types of interventions are stated above. The duration of intervention ranged from 4 to 12 weeks. The dosage of TP varied from 0.2 ug/kg/d to 1.8 g/kg/d; 0.2 mg/kg/d was the commonly administered dose.

**TABLE 1:** Characteristics of the studies included in the review.

Included studies (author, year)	Species(Sex, n = experimental/control group)	Weight (g)	Method of modeling	The criteria for modeling successfully	Type of intervention	Dosage (mg/kg/d)	Intervention duration (week)	Outcomes
An et al. (2017)	SD rats (male 20/9)	225 ± 25	HFD (4w)+STZI (60 mg/kg)	1. FBG>16.7 mmol/L (72 h of STZI) 2. Symptoms such as obvious polyuria (72 h of STZI)	T: TG C: SG	0.2/0.4	4	1. 24-h urinary albumin
Dong et al. (2017)	SD rats (male 24/12)	225 ± 25	HFD (4w)+STZI (60 mg/kg)	1. FBG>16.7 mmol/L (72 h of STZI) 2. Symptoms such as obvious polyuria (72 h of STZI)	T: TG C: SG	0.2/0.4	4	1. 24-h urinary protein
Fan et al. (2018)	C57BL/6-Ins2Akita (male 24/8)	NM	/	NM	T: TG C: SG	0.025/0.050/0.100	8	1. 24-h urinary albumin 2. SCr 3. BUN
Gao et al. (2009)	BKS db/db diabetic mice (male: female = 1:1 36/18)	NM	/	NM	T: TG C: SG	0.025/0.050	12	1. 24-h urinary albumin
Guo et al. (2016)	SD rats (male 45/15)	Unavailable data from the graph	HFD (4w)+STZI (30 mg/kg)	1. Rats with blood glucose levels>16.7 mmol/L	T: TG C: distilled water	6/12/24	4	1. 24- h urinary albumin 2. BUN
Han et al. (2017)	SD rats (male 10/10)	170 ± 10	HFD (6w)+STZI (30 mg/Kg/d, 3 days)	1. FBG>16.7 mmol/L (72 h of STZI) 2.24 h UMA levels>30 mg (2 weeks of DME)	T: TG C: SG	0.10	12	1. 24-h urinary albumin 2. SCr 3. BUN
Han (2018)	SD rats (male 10/10)	170 ± 10	HFD (8w)+STZI (30 mg/kg)	1. Random blood glucose levels of 2 or more times> 16.7 mmol/L (1 week of STZI) 2. Obvious increase of UMA level compared to NC group (6 weeks of DME)	T: TG C: DMSO	0.10	12	1. 24-h urinary albumin 2. SCr 3. BUN
Li et al. (2013)	SD rats (male 8/8)	200 g ± 20	HFD (8w)+STZI (30 mg/kg)	1. Blood glucose levels ≥16.7 mmol/L (1 week of STZI) 2.ISI ≤ ALN (1 week of STZI)	T: TG C: SG	0.2	8	1. UACR 2. SCr 3. BUN
Li et al. (2015)	SD rats (male 15/15)	230 g ± 20	HFD (4w)+STZI (30 mg/kg)	1. Random blood glucose levels ≥16.7 mmol/L (5 days of STZI)	T: TG C: edible vegetable oil	0.2	4	1. 24-h urinary albumin
Li et al. (2017)	SD rats (male 11/12)	NM	HFD (8w)+STZI (30 mg/kg)	1. Random blood glucose levels ≥16.7 mmol/L (72 h of STZI) 2.24 h UMA levels ≥30 mg (NM)	T: TG C: SG	0.2	12	1. 24-h urinary albumin 2. SCr 3. BUN
Liu et al. (2014)	SD rats (male 8/8)	190 ± 10	STZI (60 mg/kg)	1. Blood glucose level ≥16.7 mmol/L (72 h of STZI)	T: TG C: SG	0.2	8	1. 24-h urinary albumin 2. SCr 3. BUN
Ma et al. (2008)	Wistar rats (male 7/7)	200 ± 20	HFD (4w)+STZI (30 mg/kg)	1. Blood glucose level ≥16.7 mmol/L (1 week of STZI) 2. Blood pressure ≥ ALN (1 week of STZI) 3. Blood lipid ≥ ALN (1 week of STZI) 4. ISI ≤ ALN (1 week of STZI)	T: TG C: SG	0.2	12	1. UACR 2. SCr
Ma et al. (2009)	Wistar rats (male 12/12)	200 ± 20	HFD (8w)+ STZI (30 mg/kg)	1. Blood glucose level ≥16.7 mmol/L (1 week of STZI) 2. Blood pressure ≥ ALN (1 week of STZI) 3. Blood lipid ≥ ALN (1 week of STZI) 4.ISI ≤ ALN (1 week of STZI)	T: TG C: NaCMC	0.10	8	1. UACR
Ma et al. (2010)	Wistar rats (14/14)	200 ± 20	HFD (8w)+ STZI (30 mg/kg)	1. FBG ≥10.0 mmol/L (1 week of STZI) 2. ISI ≤ ALN (1 week of STZI)	T: TG C: NaCMC	0.2	8	1. UACR 2. SCr
Ma et al. (2013)	Wistar rats (male 12/11)	200 ± 20	HFD (8w)+ STZI (30 mg/kg)	NM	T: TG C: DMSO	0.1	8	1. UACR 2. SCr 3. BUN
Ma et al. (2009)	Wistar rats (male 14/14)	200 ± 20	HFD (8w)+ STZI (30 mg/kg)	1. FBG ≥10.0 mmol/L (1 week of STZI) 2. ISI ≤ ALN (1 week of STZI)	T: TG C: DMSO	0.2	8	1. 24-h urinary albumin 2. SCr
Ren et al. (2020)	BKS-db/db mice (male 6/6)	38.25 ± 1.42	/	NM	T: TG C: SG	0.05	8	1. 24-h urinary albumin 2. Plasma Cr
Wang et al. (2013)	SD rats (male 16/16)	200 ± 20	HFD (8w)+ STZI (30 mg/kg)	1. Blood glucose levels ≥16. 7 mmol/L (1 week of STZI) 2. ISI ≤ ALN (1 week of STZI)	T: TG C: SG	0.2	8	1.UACR 2.Scr 3.BUN
Wang and Yu (2017a)	SD rats (male 10/10)	190 ± 20	STZI (65 mg/kg)	1. Blood glucose levels ≥16. 7 mmol/L (1 week of STZI) 2. Glycosuria levels ≥ +++ (three consecutive days after 1 week of STZI)	T: TG C: SG	0.2	8	1.24 h urinary protein 2.Scr
Wang and Yu (2017b)	SD rats (male 10/10)	190 ± 20	STZI (65 mg/kg)	1. Blood glucose levels ≥16. 7 mmol/L (1 week of STZI) 2. Glycosuria levels ≥ +++ (three consecutive days after 1 week of STZI)	T: TG C: SG	0.2	8	1.24 h urinary protein 2.Scr
Wang and Yu (2017c)	SD rats (male 10/10)	190 ± 20	STZI (65 mg/kg)	1. Blood glucose levels ≥16. 7 mmol/L (1 week of STZI) 2. Glycosuria levels ≥ +++ (three consecutive days after 1 week of STZI)	T: TG C: SG	0.2	8	1. 24-h urinary protein 2. SCr
Xue et al., 2012	Wistar rats (male 9/7)	200 ± 20	HFD(8w) + STZI(30mg/Kg)	1. Blood glucose levels ≥10.0 mmol/L (1 week of STZI) 2. ISI ≥ ALN (1 week of STZI)	T:TG C:DMSO	0.2	8	1. 24 h urinary protein 2. SCr
Chen et al. (2018)	SD rats (male 15/15)	160 ± 8	HFD (8w)+ STZI (30 mg/kg)	1. Random blood glucose levels of 2 or more times> 16.7 mmol/L (1 week of STZI)	T: TG C: SG	0.2	12	1. 24-h urinary albumin 2. SCr 3. BUN
Ye and Hong (2015)	SD rats (male 44/22)	NM	STZI (60 mg/kg)	1. Blood glucose levels ≥16.7 mmol/L (72 h of STZI) 2. Glycosuria levels ≥ +++ (three consecutive days after 72 h of STZ injection)	T: TG C: SG	0.2	12	1. 24-h urinary albumin
You et al. (2015)	SD rats (male 13/13)	170 ± 10	STZI (52 mg/kg/d, 5 days)	1. Blood glucose levels ≥16.7 mmol/L (72 h of STZI) 2. Glycosuria levels ≥ +++ (72 h of STZI)	T: TG C: drinking water	NM	8	1. 24-h urinary albumin
Zhu (2013)	SD rats (male 44/22)	180 ± 20	STZI (60 mg/kg)	1. Blood glucose levels ≥16.7 mmol/L (72 h of STZI) 2. Glycosuria levels ≥ +++ (three consecutive days after 72 h of STZ injection)	T: TG C: SG	0.2/0.4	12	1. 24-h urinary albumin 2. SCr 3. BUN
NM: no mentioned	HFD: high fat diet	STZI: STZ injection	FBG: fasting blood glucose	ALN: the average levels of normal animals	TG: triptolide gavage	SG: 0.9% saline gavage	DME: diabetes model established	
Dong et al. (2017)	SD rats (male 12/12)	225 ± 25	HFD (4w)+STZ (60 mg/kg)	1. FBG>16.7 mmol/L (72 h) 2. Symptoms such as obvious polyuria (72 h of STZI)	T: TG P: benazepril hydrochloride	T:0.2/0.4 P:10	4	1. 24-h urinary protein
An et al. (2017)	SD rats (male 10/10)	225 ± 25	HFD (4w)+STZI (60 mg/kg)	1. FBG>16.7 mmol/L (72 h of STZI) 2. Symptoms such as obvious polyuria (72 h of STZI)	T: TG P: benazepril hydrochloride	T:0.2/0.4 P:10	4	1. 24-h urinary albumin
Gao et al. (2009)	db/db diabetic mice (male:female = 1:1 18/18)	NM	/	NM	T: TG P: valsartan	T:0.025/0.050 P:20	12	1. 24-h urinary albumin
Li et al. (2013)	SD rats (male 8/8)	200 g ± 20	HFD (8w)+STZ (30 mg/kg)	1. Blood glucose levels ≥16.7 mmol/L (1 week of STZI) 2. ISI ≤ ALN (1 week of STZI)	T: TG P: irbesartan	T:0.2 P:50	8	1. UACR 2. SCr 3. BUN
Ma et al. (2009)	Wistar rats (male 14/14)	200 ± 20	HFD (8w)+ STZ (30 mg/kg)	1. FBG ≥10.0 mmol/L (1 week of STZI) 2. ISI ≤ ALN (1 week of STZI)	T: TG P: irbesartan	T:0.2 P:50	8	1. 24-h urinary albumin 2. SCr
Ren et al. (2020)	BKS-db/db mice (male 6/6)	38.25 ± 1.42	NM	NM	T: TG P: telmisartan	T:0.050 P:5	8	1. 24-h urinary albumin 2. Plasma cr
NM: no mentioned	HFD: high-fat diet	STZI: STZ injection	FBG: fasting blood glucose	ALN: the average levels of normal animals	TG: triptolide gavage	SG: 0.9% saline gavage		

### Risk of Bias of the Studies Included

**FIGURE 2:**
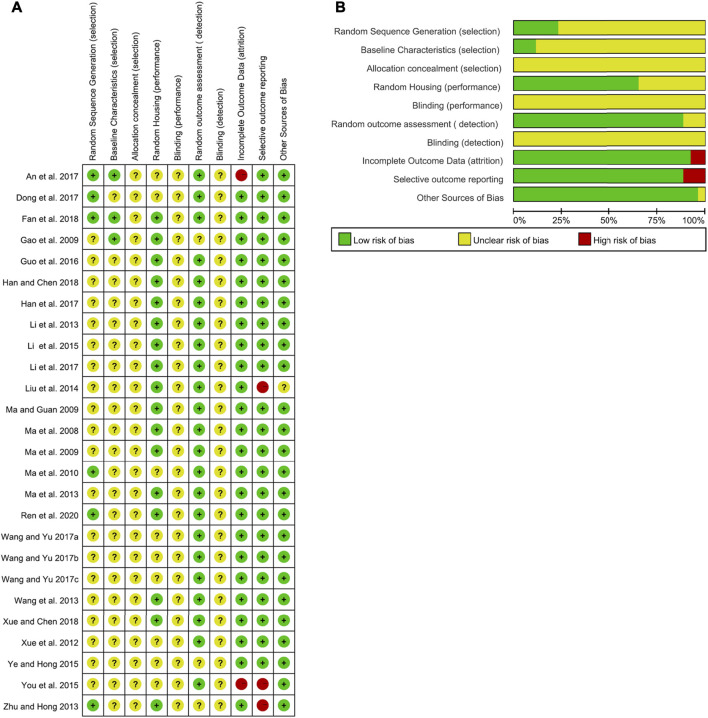
Risk of bias of the included studies. **(A)** Summary of the risk of bias. **(B)** Summary of the risk-of-bias assessment.

According to SYRCLE’s risk of bias tool for animal studies, two researchers (MH and RF) independently evaluated all included studies. The detailed results of the analyses of bias are shown in Figures 2A,B. No studies fulfilled all methodological criteria that were analyzed. Regarding selection bias, random sequence generation processes were not reported clearly in 76.92% of studies (*n* = 20). In terms of the animals’ baseline characteristics, 23 studies (88.46%) did not report this information. The unclear risks of the bias found in the studies were related to allocation concealment, blinding of caregivers, and/or investigators or outcome assessors. Nine studies (34.62%) showed incomplete information regarding random housing. Three studies (11.54%) reported unclear arbitrary outcome assessment of detection bias for relevant outcome measures. Two studies (7.70%) presented a high risk for incomplete outcome data, while three (11.54%) for selective outcome reporting. Only one research (3.85%) suggests other potential sources of bias.

### Effects of TP in Treating DKD

#### Effects on the Changes of Albuminuria, Urine Albumin/Urine Creatinine Ratio (UACR), or Proteinuria

**FIGURE 3:**
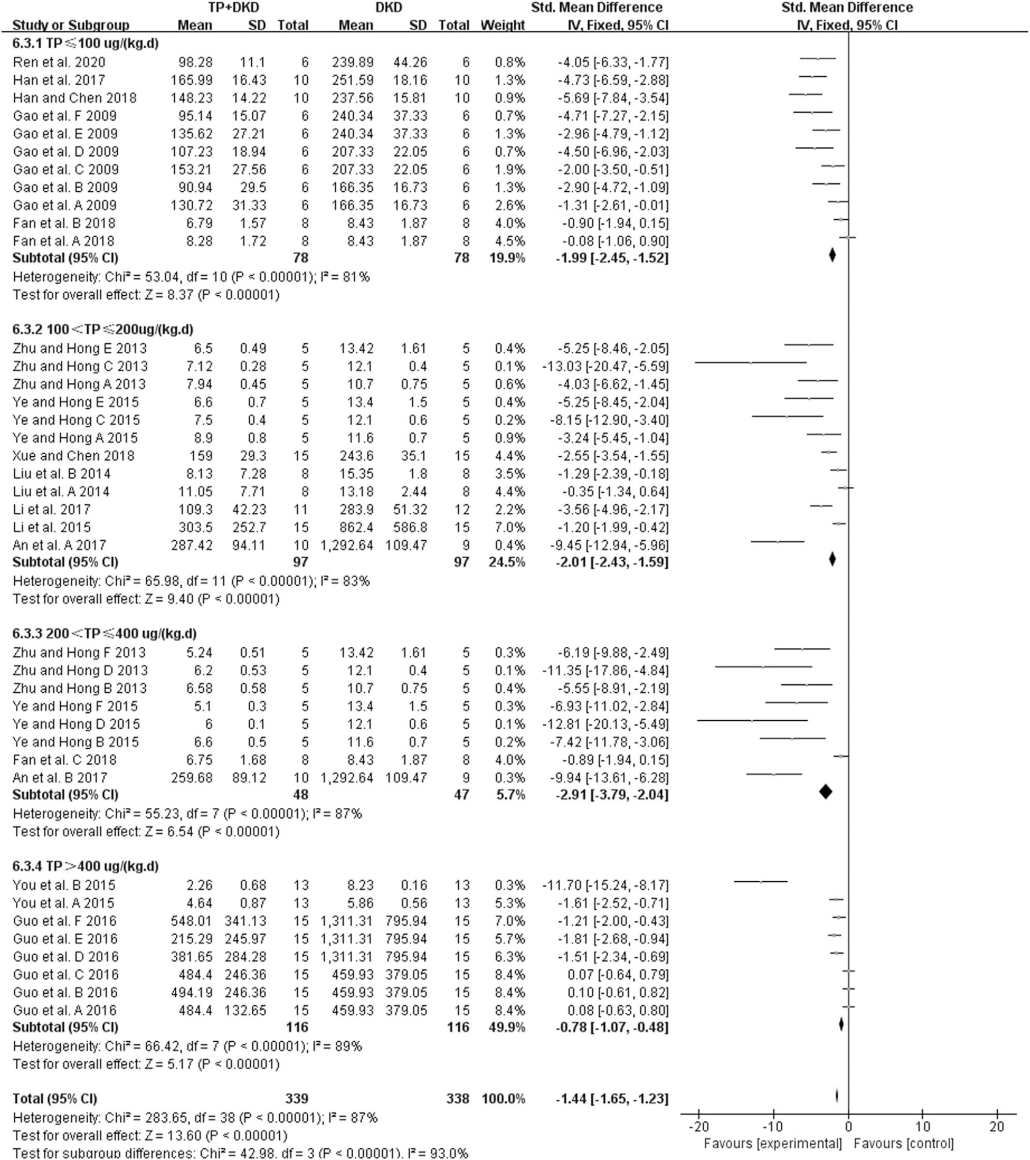
Forest plot of albuminuria outcome.

The change in albuminuria was measured in 14 studies (Qao et al., 2009; Zhu, 2013; Liu et al., 2014; Li et al., 2015; Ye and Hong et al., 2015; You et al., 2015; Guo et al., 2016; An et al., 2017; Han et al., 2017; Li et al., 2017; Fan et al., 2018; Han, 2018; Xue, 2018; Ren et al., 2020). The pooled estimation indicated that TP reduced albuminuria significantly (SMD: −1.44 [−1.65, −1.23], I^2^ = 87%), albeit with substantial heterogeneity (Figure 3).

Because of the heterogeneity associated with these studies, we performed the subgroup analyses of treatment duration (*p* < 0.00001), the dosage of triptolide (*p* = 0.002), method of modeling (*p* = 0.0003), and species of modeling (*p* = 0.85). Moreover, the meta-regression results showed that there was a linear relationship between the effect and low doses of TP. The studies in which dosage was more significant than 400 μg/kg/d were considered the high-dosage subgroup, whereas others were considered the low-dosage subgroup. The sensitivity analysis found no significant changes.

**FIGURE 4:**
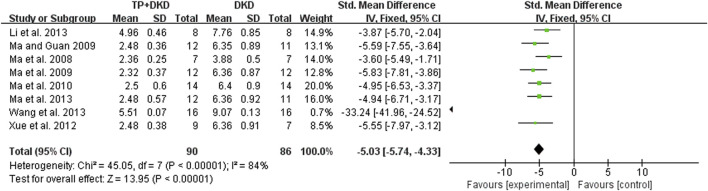
Forest plot of UACR outcome.

**FIGURE 5:**
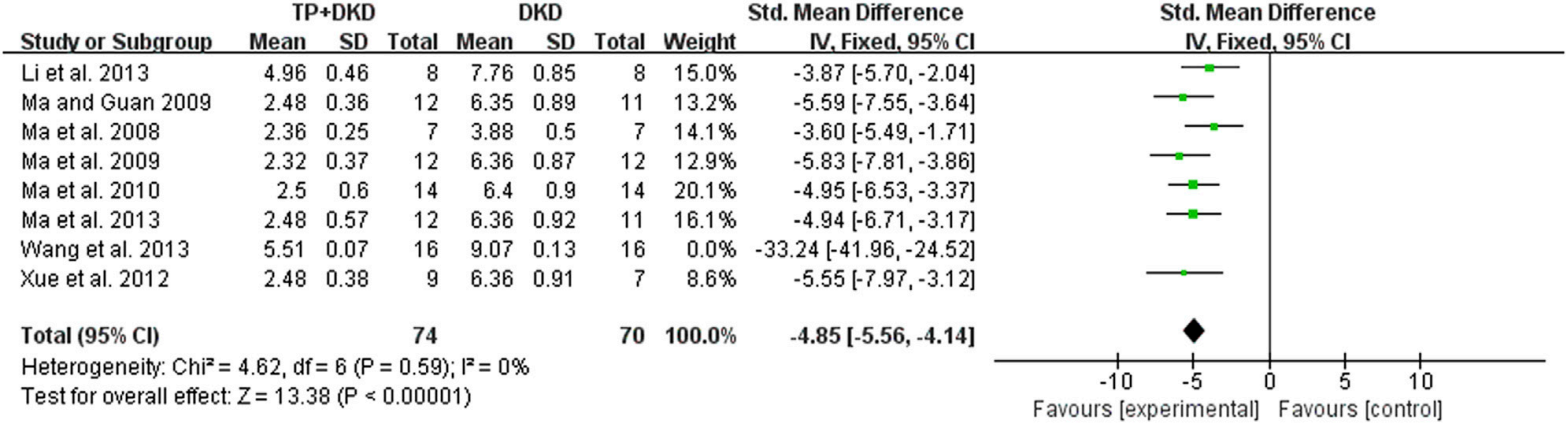
Forest plot of UACR outcome (not included by Wang et al., 2013).

Eight studies (Li et al., 2013; Ma et al., 2008; Ma, 2009; Ma et al., 2009; Ma et al., 2010; Ma et al., 2013; Wang et al., 2013; Xue et al., 2012) examined the UACR. The outcome was −5.03 mg/mg (95% CI [−5.74, −4.33]), though heterogeneity was significant (I^2^ = 84%, Figure 4). Through sensitivity analysis, it is found that heterogeneity was significantly reduced when Wang’s study (Wang et al., 2013) was eliminated (the I^2^ value reduced from 84% to 0) (Figure 5).

**FIGURE 6:**
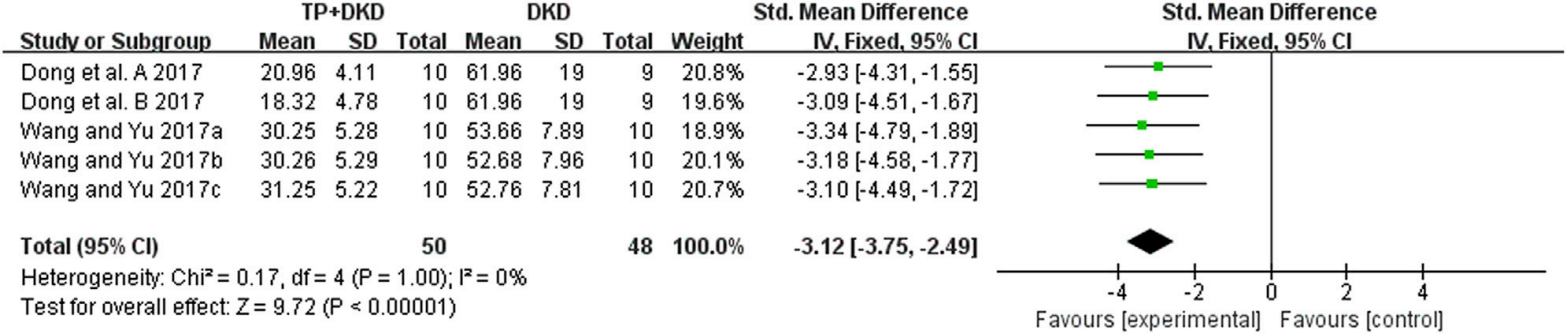
Forest plot of UP outcome.

As for proteinuria, the meta-analysis result of the four studies (Dong et al., 2017; Wang and Yu, 2017a; Wang and Yu, 2017b; Wang and Yu, 2017c) also suggested that it lowered the level of proteinuria. The I^2^ value was less than 50% (Figure 6). Since the study (Dong et al., 2017) contained two groups, we divided it into Dong et al., 2017-A and -B in this meta-analysis.

#### Effects on Kidney Function Changes

**FIGURE 7:**
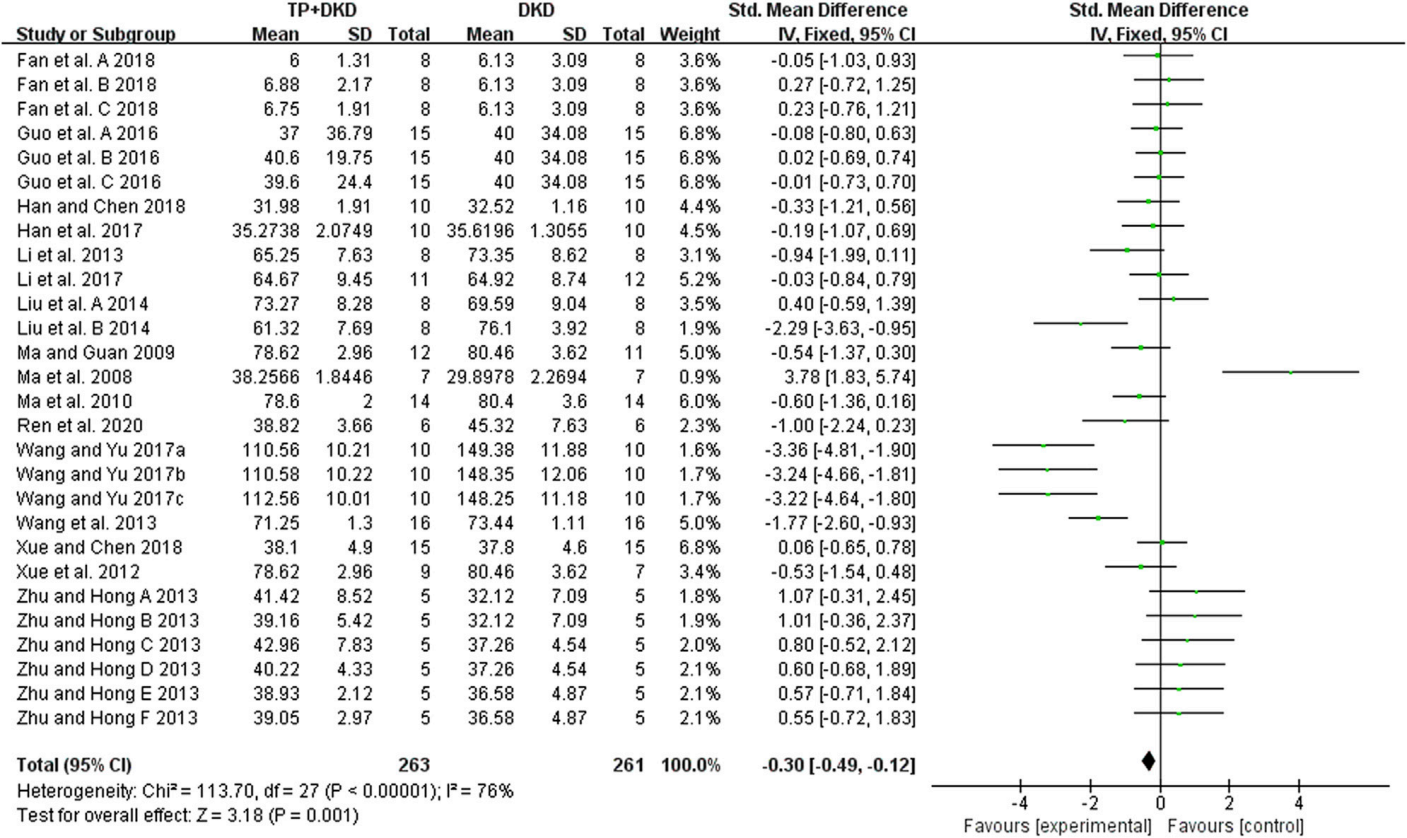
Forest plot of SCr outcome.

The kidney function was reflected by measuring the concentration of serum creatinine (SCr) and blood urea nitrogen (BUN) in the included studies. The pooled result of 18 studies (Fan et al., 2018; Guo et al., 2016; Han et al., 2017; Han, 2018; Liu et al., 2014; Li et al., 2013; Li et al., 2017; Ma et al., 2008; Ma, 2009; Ma et al., 2010; Ren et al., 2020; Wang et al., 2013; Wang and Yu, 2017a, 2017b, 2017c; Xue et al., 2012; Xue, 2018; Zhu, 2013) showed that TP had a positive effect on reducing SCr levels, with an SMD (and 95% CI) of −0.30 [−0.49, −0.12](Figure 7). The study (Fan et al., 2018) contained three groups. We then divided it into Fan et al., 2018-A, and -B, and -C in this meta-analysis. Although these results showed high heterogeneity (I^2^ value= 76%), no outliers were identified by using the sensitivity analysis. Neither treatment duration nor the dosage of TP in subgroup analyses showed differences in SCr levels.

**FIGURE 8:**
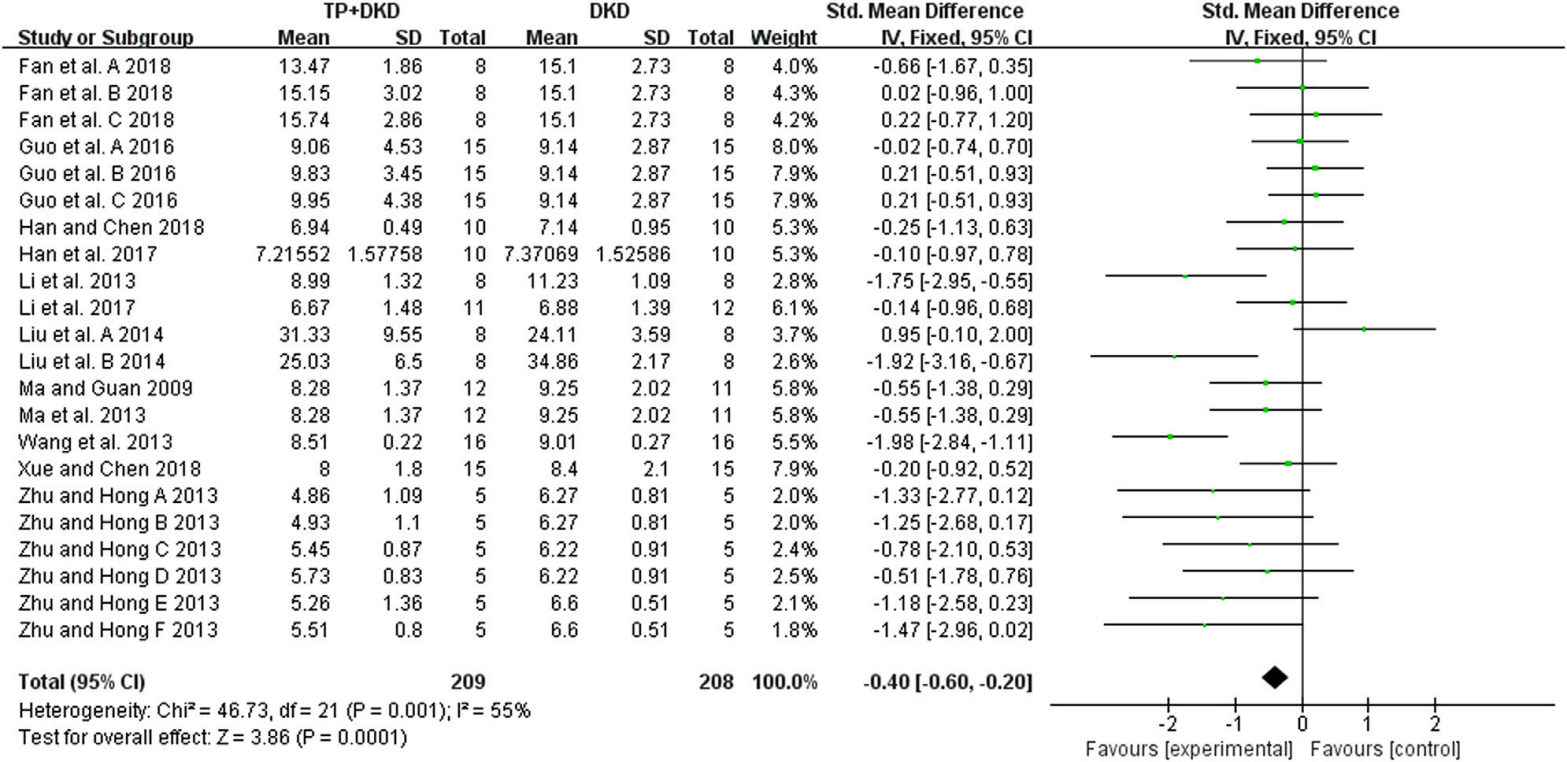
Forest plot of BUN outcome.

The BUN levels were examined in 12 studies (Ma, 2009; Li et al., 2013; Ma et al., 2013; Wang et al., 2013; Zhu, 2013; Liu et al., 2014; Guo et al., 2016; Han et al., 2017; Li et al., 2017; Fan et al., 2018; Han, 2018; Xue, 2018) (Figure 8). The meta-analysis results showed that the performance of TP was excellent in reducing BUN levels (SMD: −0.40 [−0.60, −0.20], I^2^ = 55%), compared to the control group.

### Comparison of TP and ACEI or ARB in Treating DKD

#### Effects on the Changes of Albuminuria, Urinary Albumin/Urine Creatinine Ratio (UACR), or Proteinuria

**FIGURE 9:**
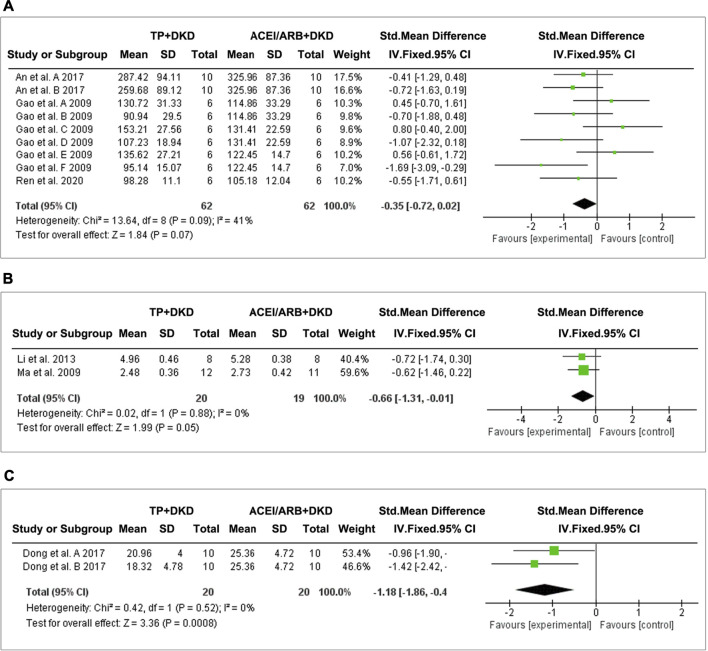
Forest plot of **(A)** albuminuria, **(B)** UACR, and **(C)** total proteinuria outcome comparing with RAS inhibitor.

We found three studies (An et al., 2017; Qao et al., 2009; Ren et al., 2020) which analyzed albuminuria (Figure 9A), two studies (Li et al., 2013, Ma et al., 2009) which analyzed the urine albumin/urine creatinine ratio (UACR) (Figure 9B), and one (Dong et al., 2017) which analyzed proteinuria (Figure 9C).

The combined results suggested no differences in albuminuria (SMD: −0.35 [−0.72, 0.02], I^2^ = 41%) between TP and ACEI or ARB. Interestingly, TP significantly reduced proteinuria (SMD: −1.18 [−1.86, −0.49], I^2^ = 0%) and the UACR (SMD: −0.66 [−1.31, −0.01], I^2^ = 0%). Due to data limitations, the reliability of this result was reduced.

#### Effects on Kidney Function Changes

**FIGURE 10:**
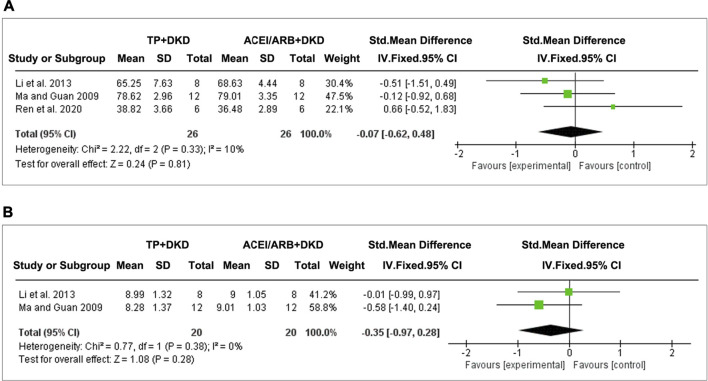
Forest plots of **(A)** SCr and **(B)** BUN outcomes compared with RAS inhibitors.

Three studies evaluated the effects of TP on SCr, and two studies assessed BUN. The pooled results implicated that, when comparing TP and ACEI or ARB, no significant differences were shown on the changes of SCr (SMD: −0.07 [−0.62, 0.48], I^2^ = 10%) (Figure 10A) and BUN (SMD: −0.35 [−0.97, 0.28], I^2^ = 0%) (Figure 10B).

### Publication Bias

**FIGURE 11:**
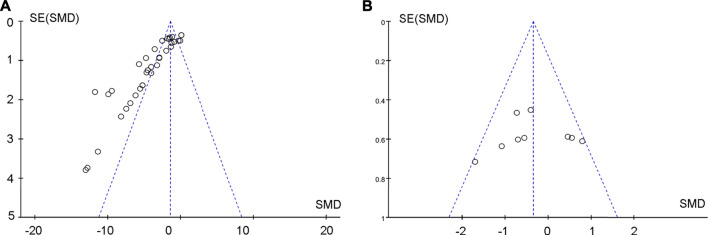
Public bias of the effects of **(A)** TP and **(B)** TP *versus* RAS inhibitors on DKD.

Begg’s and Egger’s tests were used to analyze albuminuria changes after treating DKD with TP. The funnel plot of the tests was asymmetric, and the outcome of *p*-value was less than 0.05, both of which indicated that there was publication bias (Figure 11A). The potential publication bias might be due to the high percentage of positive results being published. There was no publication bias for the effects on albuminuria between TP and ACEI or ARB in treating DKD (Figure 11B).

## Discussion

Currently, there are no effective treatments to halt DKD in the clinic which is a global health concern. The data on comparing the efficacy and safety of the current DKD therapeutic interventions remain lacking. Herein, we performed a systematic review by including 26 studies to analyze the effects of TP, an extract from a traditional Chinese herb, in treating DKD. We collected the majority of therapeutic parameters used in DKD diagnosis or clinical response evaluation, including albuminuria, proteinuria, UACR, SCr, and BUN. Serving as a characteristic indicator of the constant deterioration of DKD (de Boer et al., 2011), albuminuria and proteinuria play a key role in renal disease progression and cardiovascular events (Lin et al., 2018). It is also a sensitive biomarker for the lesions caused by DKD (Guh, 2010). As for early DKD screening (McGrath and Edi, 2019), UACR, a preferred measure of albuminuria (Sumida et al., 2020), is recommended to be detected routinely in diabetic patients who have the potential risk of renal impair (Association, 2019). In addition, to maximize the sensitivity of screening tests, SCr (Kramer, 2004) and BUN (Xie et al., 2018) are recommended as effective indicators, associating closely in renal function assessment (Zhuang et al., 2020). Under the intervention of TP, the assessment of its efficacy will be best done with the comprehensive analyzation toward the floating of the parameters above.

Our study found that TP markedly decreases proteinuria and albuminuria in DKD animal models, consistent with the previous report (Yuan et al., 2019). Since the 24-hr urinary albumin showed a high heterogeneity (χ^2^ = 283.65, I^2^ = 87%, Figure 3), we further analyzed TP effects by setting up different subgroups according to the dosages and the duration of TP treatment. Although the heterogeneity remains high, the result did exhibit significant differences (χ^2^ = 42.98, *p* < 0.00001, I^2^ = 93.0%, Figure 3) among the subgroups. According to meta-regression analysis in the low-dose subgroups (TP ≤ 400 ug/kg/d), the anti-albuminuric effects were enhanced with increasing dosage. In contrast, such effects declined in the high-dose subgroups (TP > 400 ug/kg/d). Similar results were not observed in the duration subgroup during TP treatment. It might be due to the functional “working window” of TP being relatively narrow. In other words, the therapeutic dose of TP is close to its toxic dose (Li et al., 2014; Fan et al., 2018).

A key finding of our analyses is that the effects of TP on reducing albuminuria have no differences compared to RAS inhibitors in DKD models. It is well known that RAS inhibitors are the cornerstone therapy of DKD. We speculate that such equivalent effects of TP and RAS inhibitors should attribute to the following mechanisms: 1) TP protects podocytes by inactivating the Toll-like receptor/NF-κB signaling pathway in diseased glomeruli which maintains the integrity of slit-diaphragm proteins such as nephrin and podocin (Ma et al., 2010; Wang et al., 2013; Ren et al., 2020). 2) TP ameliorates inflammation by regulating the balance of T-helper cells and repressing macrophage infiltration (Zhu, 2013; Guo et al., 2016). 3) TP alleviates oxidative stress by downregulating the renal cortex oxidative carbonyl protein and nitrotyrosine (Dong et al., 2017). 4) TP reduces glomerular mesangial cell proliferation by inactivating the PDK1/AKT/mTOR pathway (Han et al., 2017). 5) TP ameliorates glomerulosclerosis by suppressing the Notch1 pathway and regulating the content of Glut-1 and Glut-4 (You et al., 2015; Han, 2018). Of note, Li reported that a combination of TP and RAS inhibitors (irbesartan) reduced albuminuria synergistically (Li et al., 2013). The involved mechanism is unclear.

TP is the major effective monomer of the mixture, tripterygium glycosides (TGs). TG is the commercialized and commonly used extract from the TWHF herb in treating primary nephritis. In contrast to TG, TP has a similar effect on decreasing proteinuria and albuminuria induced by nephritis and DKD (Li et al., 2019). Specific to DKD intervention, the advantages of TP lies in its precision. However, a major concern regarding TP in clinical use is its multi-organ toxicity and the narrow therapeutic window (Li et al., 2014; Liu et al., 2019; Xu et al., 2019; Li et al., 2020). TP is the most important ingredient that leads to toxicity (Li et al., 2015). Primarily, triptolide is eliminated through hepatic and renal pathways. It has been revealed that the induction or inhibition of CYP3A played an important role in TP-induced hepatotoxicity (Shen et al., 2014). In addition to CYP-mediated metabolism, P-glycoprotein also played an important role in the disposition of TP and TP-induced hepatotoxicity (Xiao-Mei et al., 2013). Furthermore, members of the cytochrome P450 protein family that are involved in fatty acid (FA) metabolism, such as CYP2E1, showed the correlation between TP and its damage in kidneys. The proteomics data further suggested that FAs were involved in TP-induced toxicity (Menglin et al., 2017). Along with the advances in technology, this shortcoming of TP has been partially solved through building innovative drug delivery systems, developing water-soluble analogs, designing combinational strategies, and inventing derivatives based on structure–activity relationships (Chen et al., 2018; Liu et al., 2019). For instance, TP-encapsulated mesoscale nanoparticles (TP-MNPs) could be delivered explicitly to diseased organs to exert their therapeutic effects (Deng et al., 2019). Impressively, triptolide aminoglycoside (TPAG) is also able to protect against renal ischemia/reperfusion injury with lower toxicity to the kidney, liver, genital system, and immune system (Qi et al., 2015). A new medication developed based on TP, 14-succinate triptolide-fragment peptide (TPS-PF-A_299-585_), attenuates the thickening of the glomerular basement membrane in a membranous nephropathic rodent model. *In vitro*, TPS-PF-A_299-585_ presents anti-inflammatory activities equivalent to those of TP in the cultured kidney epithelial cells after incubation with lipopolysaccharides (Yuan et al., 2015). Intriguingly, a low-dose 14-succinyl triptolide-lysozyme (TPS-LZM) significantly hampered the progression of renal ischemia/reperfusion, whereas triptolide or lysozyme could not functionally work individually (Zhang et al., 2009). These renovations are promising and likely to be translated into DKD treatment in the clinical setting. In addition, a combinational prescription of TP, catalpol, and Panax notoginseng saponins markedly attenuates hepatotoxicity induced by TP (Zhou et al., 2018). It should be pointed out that it is a principle used to improve herbal effects and decrease its toxicity by regulating the formula in Chinese medicine. Therefore, the concrete drug–drug interactions need to be further explored.

A major issue in our current study is the high heterogeneity of the effect size of 24-h urinary albumin in DKD. At the subgroup layer, a remarkable difference (χ2 = 16.07, *p* = 0.0003, I^2^ = 87.6%) was present among subgroups of modeling. At present, there are no ideal experimental models that show all characteristics of DKD in humans. Different animal models or backgrounds could easily cause heterogeneity. Interestingly, no statistical difference (χ2 = 0.04, *p* = 0.85, I^2^ = 0%) was shown among the subgroups of strains (groups of genetic DN animal models and a group of Sprague–Dawley rats). Furthermore, our study has difficulties evaluating the risk of bias because the experiment information is lacking in some studies. Moreover, potential publication bias is shown (Figure 11). It suggests that negative outcomes are published rarely. Also, the data of certain indexes are inadequate, and the side effect profiles and adverse events are absent. With regard to this, the effects of TP might be overestimated. We should be cautious in interpreting the results.

Specific to the sensitivity analysis of UACR (Figures 4, 5), Wang’s study is considered as the main factor causing high heterogeneity. Based on the full text of the article, the composition and duration of high-fat diets, streptozotocin (STZ) administration, the timing of TP treatment, and weight of animals were similar to others except Ma et al., 2008. A significant difference was that SD rats were used by Li et al., 2013 and Wang et al., 2013, whereas Wistar rats were used by others. Marques *et al.* found that most metabolic effects, such as hyperleptinemia and decreased oral glucose tolerance, created by a high-fat diet revealed themselves earlier or more prominently in Wistar rats rather than SD rats, although the influence caused by the high-fat diet were generally alike in both strains (Marques et al., 2016). In comparison, the studies conducted by Li et al., 2013 and Wang et al., 2013, which utilized SD rats, have a slightly higher UACR in DKD groups than other studies. It is unclear whether the impact of STZ injection or the treatment of TP is different in those two strains. However, the remaining studies seemed to be a homogeneous group after removing the study conducted by Wang et al., 2013. Compared to others, Wang’s study had a larger sample size and presented a more advanced level of UACR with a minor standard deviation in both DKD and TP groups. Accordingly, it had a larger standardized mean difference effect size and confidence interval. Consequently, we ascribed the prominent effects of the study by Wang et al., 2013 to the better administration or careful treatment in the entire experiment. In addition, the standards of successfully established DKD models were different among studies included. The factors mentioned above result in multiple metabolic conditions and kidney lesions causing heterogeneity. Pooled effect sizes showed that TP decreased SCr and BUN slightly with lower heterogeneity.

There are some limitations in this review. First, as only two studies creating the group of TP combined with an RAS inhibitor, we did not have enough data to appraise the effects of this medication—co-administration in DKD models. In addition, the high heterogeneity of various factors resulted in subgroup analysis on dosage, and the treatment duration of triptolide failed to reduce it. Significant differences were caused by discordance in strains, methodologies, and criteria for modeling, stages of disease, dosages and durations of intervention, and even experiment management in each trial. It is hard to avoid it now, since there is no agreement on the establishment of animal models of DKD yet. Therefore, differences in dose, formula, and duration remain existent in the included studies in which DKD was totally induced by high-fat dietary intervention plus streptocizin injection. Furthermore, the potential bias and comparatively small studies resulted in overrated curative powers. Thus, the conclusion should be interpreted and generalized carefully. More preclinical trials with rigorous designing need to be performed to strengthen the evidence in the future.

## Conclusion

Triptolide exhibits similar pharmacological effects to RAS inhibitors on reducing albuminuria and preserving renal function after the onset of DKD. Although there was data heterogeneity, this meta-analysis result provides the clinicians with potential options in designing interventional strategies for DKD patients. Meanwhile, the renovations on TP derivatives and drug-delivering systems are believed to be promising to shed light on DKD prevention in the future.

## Data Availability

The original contributions presented in the study are included in the article/Supplementary Material; further inquiries can be directed to the corresponding author.
